# Phosphorylated Alpha-Synuclein and Carboxymethyllysine in the Epidermis of Type 2 Diabetes Patients: Preliminary Observations

**DOI:** 10.3390/biomedicines14051127

**Published:** 2026-05-16

**Authors:** Bernard Kordas, Wojciech Matuszewski, Robert Modzelewski, Judyta Juranek

**Affiliations:** 1 Department of Human Physiology and Pathophysiology, School of Medicine, Collegium Medicum, University of Warmia and Mazury, 10-082 Olsztyn, Poland; 2 Clinic of Endocrinology, Diabetology and Internal Medicine, School of Medicine, Collegium Medicum, University of Warmia and Mazury, 10-561 Olsztyn, Poland

**Keywords:** alpha-synuclein, carboxymethyllysine, diabetes, advanced glycation end-products, glycation

## Abstract

**Background/objectives**: Alpha-synuclein (aSyn) is best known for its role in Parkinson’s disease. Increasing evidence suggests a bidirectional relationship between diabetes mellitus and synuclein pathology. Carboxymethyllysine (CML), an advanced glycation end-product, serves as a marker of cumulative glycation stress and tissue damage in diabetes. Our study aimed to evaluate epidermal phosphorylated alpha-synuclein at Ser129 (p-aSyn) immunoreactivity in relation to CML accumulation in epidermis. **Methods:** Skin punch biopsies were obtained from seven diabetic patients with long-standing type 2 diabetes (T2DM), and from seven healthy volunteers. Tissue samples were processed and analyzed by immunohistochemical DAB-staining for p-aSyn and CML. Quantitative analysis was performed by measuring the percentage area of positive staining using Fiji/ImageJ2. Integrated density was also assessed as a complementary threshold-limited measure of staining signal intensity. Statistical analysis and data visualization were conducted using GraphPad Prism. Comparisons between groups were performed using the exact two-tailed Mann–Whitney U test. **Results**: Area-fraction analysis showed significantly greater CML-positive staining in diabetic epidermis than in controls (median 10.18 vs. 8.955, *p* = 0.0262), whereas p-aSyn-positive area fraction did not differ significantly between groups (13.53 vs. 14.64, *p* = 0.8048). In the complementary integrated density analysis, p-aSyn signal was significantly higher in diabetic epidermis than in controls (21,365 vs. 10,960, *p* = 0.0023), whereas the increase in CML integrated density did not reach statistical significance (14,165 vs. 6585, *p* = 0.1282). In diabetic epidermis, both markers showed a more widespread distribution, involving basal keratinocyte cytoplasm and extension into suprabasal layers. Control samples showed staining largely restricted to basal cell contours. In serial sections, p-aSyn and CML showed a similar topographic distribution in diabetic skin. **Conclusions**: These preliminary observations suggest that chronic diabetic skin changes are associated with increased epidermal CML burden when assessed by area fraction and with higher p-aSyn signal intensity when assessed by integrated density. However, because the study was small and based on semiquantitative DAB immunohistochemistry, the findings should be interpreted cautiously and require validation in larger multimodal studies.

## 1. Introduction

Alpha-synuclein (aSyn), best known for its role in the pathogenesis of Parkinson’s disease (PD) and other synucleinopathies, has recently attracted attention as a potential modulator of glucose homeostasis and a key target of protein glycation in both diabetic and PD patients [1]. Aggregated α-synuclein is a major component of Lewy bodies and Lewy neurites, although Lewy pathology also contains lipid membranes, vesicular structures, and dysmorphic organelles [2,3]. Traditionally regarded as a neuronal protein, increasing evidence indicates that aSyn is widely expressed in peripheral tissues, including pancreatic islets, skeletal muscle, and the skin, suggesting a physiological role beyond the central nervous system [4,5].

Recent studies have demonstrated that aSyn deficiency impairs glucose metabolism and increases the risk of developing insulin resistance linked to metabolic syndrome and diabetes [6]. Patients with type 2 diabetes mellitus (T2DM) show an increased risk of developing PD, while individuals with PD frequently exhibit impaired glucose tolerance and insulin resistance [6,7,8]. Experimental models demonstrate that loss of aSyn results in glucose intolerance and insulin resistance, whereas increased aSyn expression improves insulin sensitivity and glucose-stimulated insulin secretion [1,9]. Protein glycation represents a key biochemical link between hyperglycemia and tissue damage in diabetes. In this context, aSyn may also be susceptible to glycation-related modification, linking metabolic stress with altered protein function.

Chronic exposure to elevated glucose levels leads to the formation of advanced glycation end-products (AGEs), which accumulate in long-lived proteins and contribute to cellular dysfunction through oxidative stress and inflammatory pathways mediated by receptor [10,11]. Among these AGEs, carboxymethyllysine (Nε-carboxymethyl-L-lysine, CML) is one of the most widely studied and stable markers of cumulative glycation stress. Elevated levels of CML in skin collagen have been shown to predict the long-term risk of diabetic microvascular complications, highlighting the importance of skin as a valuable tissue for assessing systemic glycation [12,13].

**Figure 1:**
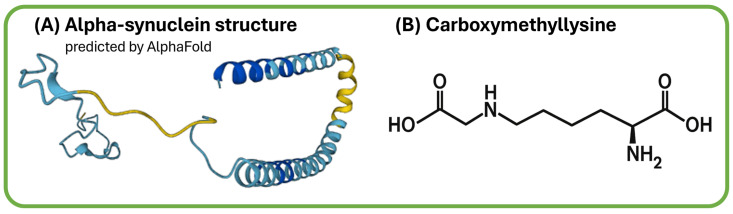
(**A**) Predicted structure of alpha-synuclein from the AlphaFold Protein Structure Database [16,17,18]. (**B**) Chemical structure of Nε-carboxymethyl-L-lysine (CML), adapted by the authors based on PubChem Compound Summary for CID 123800 [19].

Multiple studies have demonstrated the presence of native and phosphorylated aSyn (p-aSyn) in cutaneous nerve fibers, keratinocytes, and dermal structures, underlying the importance of utilizing skin biopsies to gain insight into systemic and neurodegenerative processes [14,15]. While most prior work has focused on p-aSyn in the context of synucleinopathies, considerably less is known about the distribution of p-aSyn in diabetic skin and its relationship to markers of chronic glycation stress, such as CML, in non-neuronal epidermal compartments. Therefore, the present study aimed to evaluate epidermal p-aSyn immunoreactivity in relation to CML accumulation in skin biopsies obtained from long-term T2DM patients and from healthy controls. Given the exploratory character of the study, the findings are intended as preliminary observations rather than as evidence of a direct mechanistic relationship between glycation and p-aSyn in the skin (Figure 1).

## 2. Materials and Methods

### 2.1. Study Participants

Seven control participants (aged 45.71 ± 4.42 years) and seven patients with T2DM (aged 43.86 ± 8.03 years, with disease duration of 15.57 ± 2.88 years), treated at the Clinic of Endocrinology, Diabetology and Internal Medicine, Department of Internal Medicine, University of Warmia and Mazury in Olsztyn, Poland, were enrolled in the study. Control participants were healthy, normoglycemic, normotensive, normolipidemic, and free of known comorbidities, with a mean HbA1c of 5.11 ± 0.29%. Patients with T2DM had a mean HbA1c of 8.50 ± 0.95%, with hypertension, dyslipidemia, and clinically confirmed distal sensory neuropathy of the lower limbs, and had no known other comorbidities unrelated to diabetes. Following diagnosis, all diabetic participants had been treated in accordance with national and European recommendations, mainly those of the Diabetes Poland (Polskie Towarzystwo Diabetologiczne, PTD) and the European Association for the Study of Diabetes (EASD), with treatment primarily including metformin, incretin-based medications, and sodium-glucose cotransporter-2 inhibitors. Only non-smokers and individuals without alcohol abuse were included in either group. The University Institutional Ethics Committee approved the study (No. 10/2010 dated 25 March 2010). The present analysis was performed within the scope of this approval. Before enrolling, all subjects provided informed consent.

### 2.2. Skin Biopsy Collection, Preparation and Immunohistochemistry

Skin biopsies of clinically intact skin were obtained under local anesthesia, using sterile disposable punch instruments, to a depth of approximately 3 mm, 10 cm above the lateral malleolus of the non-dominant limb. The biopsies were transferred to 4% paraformaldehyde (pH 7.4) for 10 min, washed in phosphate buffer and immersed in 20% sucrose solution for cryoprotection at 4 °C until they sank to the bottom of the individual 2 mL vials, before further processing.

Samples were then embedded in Tissue-Tek O.C.T. Compound (Sakura Finetek, Torrance, CA, USA), frozen at −24 °C in a cryostat (Hyrax C25, Zeiss, Oberkochen, Germany), and cryosectioned at −24 °C. Frozen samples were cut into 10 µm sections, air-dried and stored frozen at −22 °C until further immunostaining. To minimize repeated sampling of the same structures, only one out of every five consecutive sections was included in the analysis, resulting in a 50 µm interval between analyzed sections.

Immunohistochemical staining was performed using primary antibodies against p-aSyn (ab51253, 1:200, Abcam, Cambridge, UK) and CML (ab125145, 1:200, Abcam, Cambridge, UK), followed by DAB (3,3′-diaminobenzidine) processing according to the manufacturer’s guidelines (Vectastain ABC-HRP kit, Vector Laboratories, Newark, CA, USA). No antigen retrieval was performed before immunohistochemical staining. Analyses were performed in batches to ensure consistency of antibody solutions and uniform staining conditions across samples. Negative control sections were also processed without antibody to assess the non-specific background signal.

### 2.3. Image Acquisition, Quantitative and Statistical Analysis

Immediately after staining, all samples were imaged using identical system acquisition settings. Samples were examined using an Olympus IX83 microscope (Evident, Tokyo, Japan), and images were acquired in a standardized manner to capture the epidermis as completely as possible and to maintain a consistent orientation across samples.

Quantitative image analysis was performed using Fiji/ImageJ2 version 2.16.0/1.54p (National Institutes of Health, Bethesda, MD, USA) [20,21]. For each analyzed image, the region of interest (ROI) included the epidermis without the stratum corneum and excluded the dermis and obvious non-tissue background when present. Bright-field RGB images were converted to 8-bit grayscale before analysis. For both markers, threshold values were established before batch quantification using negative control sections as a reference for background signal and were then kept unchanged for all control and diabetic images. The threshold range was set from 0 to 100 grayscale units for both p-aSyn and CML. Area fraction of positive staining was then measured within the manually defined epidermal ROI. In addition, integrated density was measured as a complementary parameter limited by threshold within the same ROI. For integrated density analysis, 8-bit grayscale images were inverted so that stronger DAB staining corresponded to higher pixel values. After inversion, pixels representing stronger staining approached 255, and weakly stained or unstained regions approached 0. This transformation allowed integrated density to be interpreted as an intensity-weighted measure of the DAB-positive signal. This approach was used to maintain consistency of quantification across control and diabetic samples. For each participant and each marker, four stained tissue sections were analyzed. Within each section, measurements were performed in three non-overlapping image fields, which served as technical replicates. The resulting 12 technical measurements were averaged to obtain one participant-level value per marker, and these values were used for statistical comparisons.

Given the small group sizes, comparisons between groups were performed using the exact two-tailed Mann–Whitney U test. Descriptive data are provided as means ± standard deviation (SD) and medians, and group differences are summarized using exact *p* values, Mann–Whitney U values, and Hodges-Lehmann estimates. In the figures, each dot represents one participant-level value, and horizontal bars indicate group means. Statistical analyses were conducted using GraphPad Prism 10 version 10.5.0 for Windows (GraphPad Software, Boston, MA, USA).

## 3. Results

**Figure 2:**
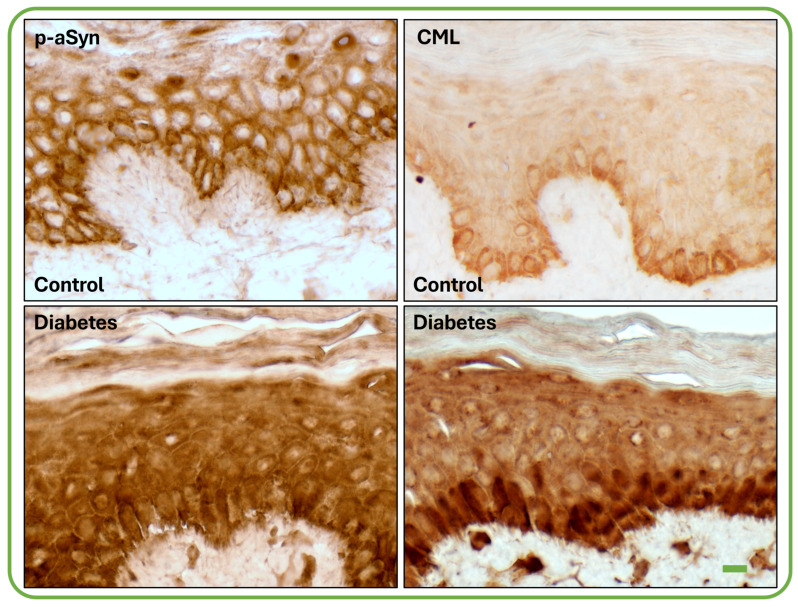
Representative images of immunohistochemical DAB staining for p-aSyn and CML in control and diabetic skin biopsies. In control epidermis, staining for both markers was mainly associated with basal cell contours. In diabetic epidermis, p-aSyn and CML immunoreactivity showed a more diffuse distribution, involving basal keratinocyte cytoplasm and extension into suprabasal layers. Images were acquired at 40× magnification. Scale bar: 20 µm for all panels.

Immunohistochemical staining for p-aSyn and CML showed distinct distribution patterns in control and diabetic epidermis. In control samples, immunoreactivity for both markers was predominantly confined to the basal epidermal layer and was most evident along cell borders and superficial cellular contours. In diabetic samples, staining for both p-aSyn and CML was more widely distributed, involving not only basal cell outlines but also the cytoplasm of basal keratinocytes, extending into suprabasal layers. Thus, although true colocalization could not be assessed in this study, CML and p-aSyn displayed similar topographic distribution in serial sections from diabetic skin (Figure 2).

CML staining in control epidermis was most prominent in the innermost layer, delineating individual basal cells. In diabetic samples, CML immunoreactivity filled the interior of basal cells and was additionally visible in cells of overlying layers and within the intercellular space. The p-aSyn staining was less intense in control samples and was mainly associated with the contours of basal and adjacent suprabasal cells. In samples from patients with diabetes, p-aSyn staining was detected both at the cell periphery and within the cells. Using area fraction as the semiquantitative endpoint, CML-positive staining occupied a significantly greater proportion of the epidermal ROI in diabetic skin than in control skin (median 10.18 vs. 8.96, exact two-tailed Mann–Whitney U = 7, *p* = 0.0262; Hodges–Lehmann estimate = 1.802). In contrast, p-aSyn-positive area fraction did not differ significantly between groups (median 13.53 vs. 14.64, U = 22, *p* = 0.8048; Hodges–Lehmann estimate = −0.623). Mean area fraction values were 11.02 ± 2.33 in diabetic versus 8.64 ± 1.23 in control epidermis for CML, and 13.71 ± 3.59 versus 14.19 ± 5.26 for p-aSyn, respectively.

**Figure 3:**
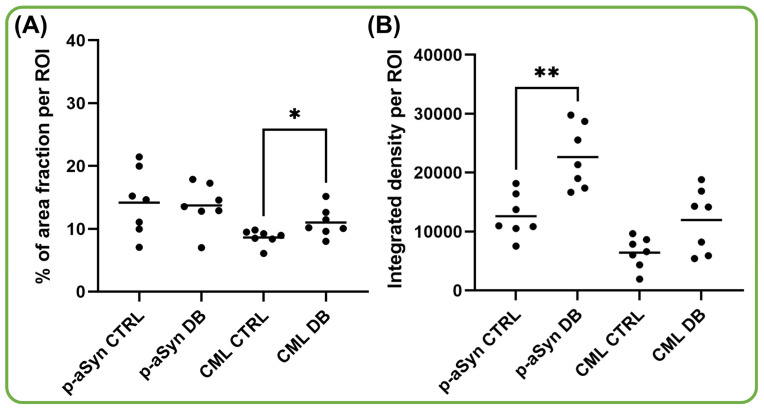
Quantitative analysis of p-aSyn and CML immunoreactivity in control and diabetic epidermis. (**A**) Area fraction per ROI. (**B**) Integrated density per ROI. Each dot represents one participant-level value; horizontal bars indicate group means. Statistical comparisons were performed using the exact two-tailed Mann–Whitney U test. In area-fraction analysis, CML differed significantly between groups (*p* = 0.0262), whereas p-aSyn did not (*p* = 0.8048). In integrated-density analysis, p-aSyn differed significantly between groups (*p* = 0.0023), whereas CML did not (*p* = 0.1282). Asterisks indicate significant comparisons shown on the graph: * *p* = 0.0262 and ** *p* = 0.0023.

In the complementary integrated density analysis restricted to the epidermal ROI using threshold, p-aSyn showed a significantly higher signal in diabetic epidermis than in controls (median 21,365 vs. 10,960, U = 2, *p* = 0.0023; Hodges–Lehmann estimate = 9869). By contrast, CML integrated density was numerically greater in diabetic samples but did not reach statistical significance (median 14,165 vs. 6585, U = 12, *p* = 0.1282; Hodges–Lehmann estimate = 6301). Mean integrated density values were 22,634 ± 5388 in diabetic versus 12,595 ± 3712 in control epidermis for p-aSyn, and 11,957 ± 5396 versus 6431 ± 2649 for CML, respectively. Quantitative analysis therefore suggested different marker-specific patterns depending on the metric used. CML differed significantly between groups when assessed by area fraction, whereas p-aSyn differed significantly when assessed by integrated density (Figure 3).

## 4. Discussion

The present study showed a significantly greater CML-positive area fraction in diabetic epidermis and a more diffuse epidermal distribution of both CML and p-aSyn immunoreactivity in diabetic skin compared with control skin. Although p-aSyn-positive area fraction did not differ significantly between groups, complementary integrated density analysis showed a significantly higher p-aSyn signal in diabetic epidermis. Accordingly, the most conservative interpretation of the present findings is that long-standing T2DM is associated with increased epidermal glycation and with differences in the intensity-weighted p-aSyn immunoreactive signal, rather than with a uniform increase across all quantitative measures. Importantly, all diabetic participants had clinically confirmed distal sensory neuropathy of the lower limbs. However, because the present study did not quantify nerve fiber pathology or neuropathy severity, these findings should be interpreted as arising in the clinical setting of diabetic neuropathy rather than as direct markers of neuropathic involvement.

An additional observation was that area-fraction and integrated density analyses yielded different statistical patterns. CML differed significantly between diabetic and control epidermis when assessed by area fraction, whereas p-aSyn differed significantly between groups when assessed by integrated density. Area fraction reflects the proportion of the ROI occupied by threshold-positive staining, and integrated density incorporates both the extent of threshold-positive staining and the intensity of signal within those pixels. Accordingly, the present data may suggest that diabetic epidermis is characterized by a greater spatial extent of detectable CML staining, while differences in p-aSyn are more apparent at the level of staining intensity than at the level of the overall fraction of stained epidermal area. However, because both measures were derived from single-marker DAB immunohistochemistry and depend on threshold-based image processing, these observations should be interpreted cautiously and regarded as semiquantitative rather than definitive evidence of molecular abundance or direct biochemical modification.

This interpretation is biologically plausible and consistent with prior work showing that skin collagen glycation and advanced glycation end-products can predict long-term progression of diabetic microvascular complications, including retinopathy and nephropathy [13,22]. Skin is also a biologically relevant target of signaling related to AGEs, and RAGE has been shown to be expressed in human skin, including keratinocytes [23]. In parallel, hyperglycemic conditions have been shown to alter keratinocyte morphology, impair glucose utilization, reduce proliferation and migration, enhance differentiation, and increase IL-8 signaling in a manner dependent on ROS [24,25,26]. Within this context, the increased CML signal and a more extensive epidermal distribution observed in our diabetic samples are consistent with chronic stress related to glycation in metabolically responsive skin tissue. This interpretation is supported by the clinical profile of the diabetic group, which remained metabolically distinct from controls despite treatment, including elevated HbA1c values, long disease duration, and clinically assessed distal sensory neuropathy.

The pattern of p-aSyn immunoreactivity in diabetic epidermis should nevertheless be interpreted with caution. Most previous skin biopsy studies of aSyn have been performed in the context of synucleinopathies, with an emphasis on cutaneous nerve fibers and other dermal structures rather than on epidermal staining in metabolic disease. Therefore, direct comparison of our findings with the established skin biopsy literature is limited. aSyn has been detected in skin in previous biopsy studies, and p-aSyn has been studied particularly in the context of synucleinopathies [14,15]. In the present work, the antibody used recognized aSyn phosphorylated at Ser129 rather than total aSyn. Accordingly, the observed signal should be interpreted as p-aSyn immunoreactivity and not as a measure of total aSyn abundance. Because the present DAB-based single-marker approach did not include nerve fiber markers, we cannot determine whether the p-aSyn signal reflected keratinocytes, intraepidermal nerve fibers, or both. The finding that p-aSyn differed between groups only when assessed by integrated density, but not by area fraction, points to the interpretation that the diabetic samples may differ mainly in signal intensity rather than in the overall proportion of epidermal area occupied by detectable p-aSyn staining. This distinction is important, as the present study was not designed to provide insight into the relationships among total aSyn, p-aSyn, aggregated species, or glycated species in diabetic skin. Rather, we aimed to provide background information on the presence of p-aSyn in the epidermis of long-term diabetic patients. Glycation has been shown to modify aSyn, impair its clearance, alter its aggregation behavior, and promote toxic or pro-inflammatory downstream effects in experimental models [27,28]. However, the present findings do not demonstrate direct glycation-dependent modification of p-aSyn in the epidermis. Rather, they indicate that increased glycation burden and differences in p-aSyn immunoreactivity may coexist in diabetic skin. Because CML and p-aSyn were evaluated in separate staining, it cannot be concluded that both markers occupy the same subcellular compartment. Thus, the serial sections only suggest a similar topographic distribution of both markers in diabetic epidermis.

The more diffuse intracellular and suprabasal staining pattern observed in diabetic samples may reflect changes in cellular handling of p-aSyn under chronic metabolic stress. However, this remains a hypothesis rather than a conclusion. The present analysis was based on DAB immunohistochemistry and area-fraction measurements, which provide semiquantitative spatial information but not molecular specificity.

Epidemiological studies associate T2DM with a higher subsequent risk of Parkinson’s disease, and that risk increases with diabetes duration and severity [29,30,31]. However, our study did not assess synucleinopathy features, intraepidermal nerve fiber pathology, or neurological outcomes, and it should not be interpreted as evidence of prodromal synucleinopathy or as a biomarker study for Parkinson’s disease. Rather, our findings support the rationale for investigating skin as an accessible tissue in which chronic metabolic stress and p-aSyn immunoreactivity may intersect.

Several limitations should be acknowledged. First, this was a small exploratory study involving a limited number of participants, which reduces statistical power. Recruitment was further constrained by the invasive nature of skin punch biopsy, particularly in patients with long-standing diabetes, in whom wound healing may be prolonged and associated with minor complications. Second, the study relied on DAB-based single-marker immunohistochemistry and threshold-based area fraction and integrated density analyses, which provide semiquantitative spatial and intensity-weighted information but not molecular specificity. Although negative controls were performed and thresholding was kept constant within each staining set, the method does not provide direct information on aggregation state, phosphorylation dynamics, or glycation of aSyn. It also does not distinguish p-aSyn immunoreactivity associated with epidermal cells from potential signal associated with intraepidermal nerve fibers. Integrated density is also sensitive to image processing, threshold selection, staining intensity, and grayscale transformation. Thus, it should be interpreted as a complementary semiquantitative measure rather than as an absolute measure of protein abundance. Third, it was a cross-sectional human study in patients with long-term diabetes, and the cumulative effects of disease duration, glycemic control, medication adherence, lifestyle measures, and concomitant treatments may have differed substantially between individuals over time. Although all patients were treated according to guidelines, these variables could not be standardized retrospectively. The diabetic cohort was intentionally studied as a clinically similar group of patients with long-standing T2DM and distal sensory neuropathy, but this does not eliminate potential confounding by comorbidities or treatment exposure. Accordingly, the present findings should be regarded as preliminary observations that require confirmation in larger studies with more detailed phenotyping and multimodal tissue analysis.

## 5. Conclusions

Diabetic epidermis showed increased CML immunoreactivity and a more extensive epidermal distribution of both CML and p-aSyn than in control tissue. CML-positive area fraction was significantly greater in diabetic epidermis, whereas p-aSyn-positive area fraction did not differ significantly between groups. In contrast, complementary integrated density analysis showed a significantly higher p-aSyn signal in diabetic epidermis, while CML integrated density did not reach statistical significance. Taken together, these preliminary observations support further studies on the relationship between chronic glycation stress and epidermal p-aSyn immunoreactivity in diabetes.

## Data Availability

The original contributions presented in this study are included in the article. The raw data supporting the conclusions of this article will be made available by the authors on request.

## Abbreviations

The following abbreviations are used in this manuscript:

AGEs	Advanced glycation end-products
aSyn	Alpha-synuclein
CML	Carboxymethyllysine
DAB	3,3′-Diaminobenzidine
EASD	European Association for the Study of Diabetes
p-aSyn	Phosphorylated alpha-synuclein
PD	Parkinson’s disease
ROI	Region of interest
SNCA	Synuclein alpha gene
T2DM	Type 2 diabetes mellitus
